# Automated Sulcal Depth Measurement on Cortical Surface Reflecting Geometrical Properties of Sulci

**DOI:** 10.1371/journal.pone.0055977

**Published:** 2013-02-13

**Authors:** Hyuk Jin Yun, Kiho Im, Jin-Ju Yang, Uicheul Yoon, Jong-Min Lee

**Affiliations:** 1 Department of Biomedical Engineering, Hanyang University, Seoul, South Korea; 2 Division of Newborn Medicine, Children’s Hospital Boston, Harvard Medical School, Boston, Massachusetts, United States of America; 3 Department of Biomedical Engineering, College of Health and Medical Science, Catholic University of Daegu, Gyeongsan-si, South Korea; Centre Hospitalier Universitaire Vaudois Lausanne - CHUV, UNIL, Switzerland

## Abstract

Sulcal depth that is one of the quantitative measures of cerebral cortex has been widely used as an important marker for brain morphological studies. Several studies have employed Euclidean (EUD) or geodesic (GED) algorithms to measure sulcal depth, which have limitations that ignore sulcal geometry in highly convoluted regions and result in under or overestimated depth. In this study, we proposed an automated measurement for sulcal depth on cortical surface reflecting geometrical properties of sulci, which named the adaptive distance transform (ADT). We first defined the volume region of cerebrospinal fluid between the 3D convex hull and the cortical surface, and constructed local coordinates for that restricted region. Dijkstra’s algorithm was then used to compute the shortest paths from the convex hull to the vertices of the cortical surface based on the local coordinates, which may be the most proper approach for defining sulcal depth. We applied our algorithm to both a clinical dataset including patients with mild Alzheimer’s disease (AD) and 25 normal controls and a simulated dataset whose shape was similar to a single sulcus. The mean sulcal depth in the mild AD group was significantly lower than controls (*p* = 0.007, normal [mean±SD]: 7.29±0.23 mm, AD: 7.11±0.29) and the area under the receiver operating characteristic curve was relatively high, showing the value of 0.818. Results from clinical dataset that were consistent with former studies using EUD or GED demonstrated that ADT was sensitive to cortical atrophy. The robustness against inter-individual variability of ADT was highlighted through simulation dataset. ADT showed a low and constant normalized difference between the depth of the simulated data and the calculated depth, whereas EUD and GED had high and variable differences. We suggest that ADT is more robust than EUD or GED and might be a useful alternative algorithm for measuring sulcal depth.

## Introduction

Many researchers in neuroimaging studies have extracted and analyzed various brain morphological features, including curvature [1]–[3], fractal dimension [4], [5], thickness [1], [2], [6], [7], gyrification index [2], [8], [9], sulcal pits [10], [11] and sulcal depth [1], [3], [9], based on the characteristics of the brain, which is a highly convoluted and folded structure. In particular, sulcal depth has been studied as an important index for cerebral health and has been widely used to study the morphology of the cortical folding [1], [3], [11]–[13]. Sulcal depth has two specific properties. First, the spatial distribution of deep sulcal regions is relatively robust against interindividual variability [10], [14], [15]. The structural deepening and folding process of sulci is related to functional areas and occurs during brain development, including early radial growth and later tangential growth. This brain developmental trajectory leads to complex sulcal geometry containing both spatial invariants and interindividual variability [10], [16]–[19]. The deep sulcal regions are thought to be the first cortical folds in the early stages during development [20], [21] and their formation might be related to genetic control and cytoarchitectonic areas [16]. Furthermore, brain morphological studies have used sulcal depth as deep sulcal landmarks such as sulcal fundi [22], lines [23] and pits [10], [11] have been extracted using a sulcal depth map. The second property is that sulcal depth is sensitive to cortical atrophy. Cortical atrophy is thought to be related to reduction in cortical thickness and gyral white matter volume [1] or tension of the cortico–cortical connections in subcortical white matter [24]. Sulcal depth has been used in several studies that analyzed the brain morphological changes caused by age-related trends, Williams syndrome, schizophrenia or Alzheimer’s disease (AD) [1], [9], [12], [24]–[26]. These studies reported generally shallower sulcal depths related to cortical atrophy or disease progression. Because cortical structures including sulci are affected by multiple influences such as genetic factors, neurological/psychiatric disorders and aging, it is important that the algorithm for sulcal depth needs to be highly sensitive to morphological change while remaining robust against sulcal variability.

**Figure 1:**
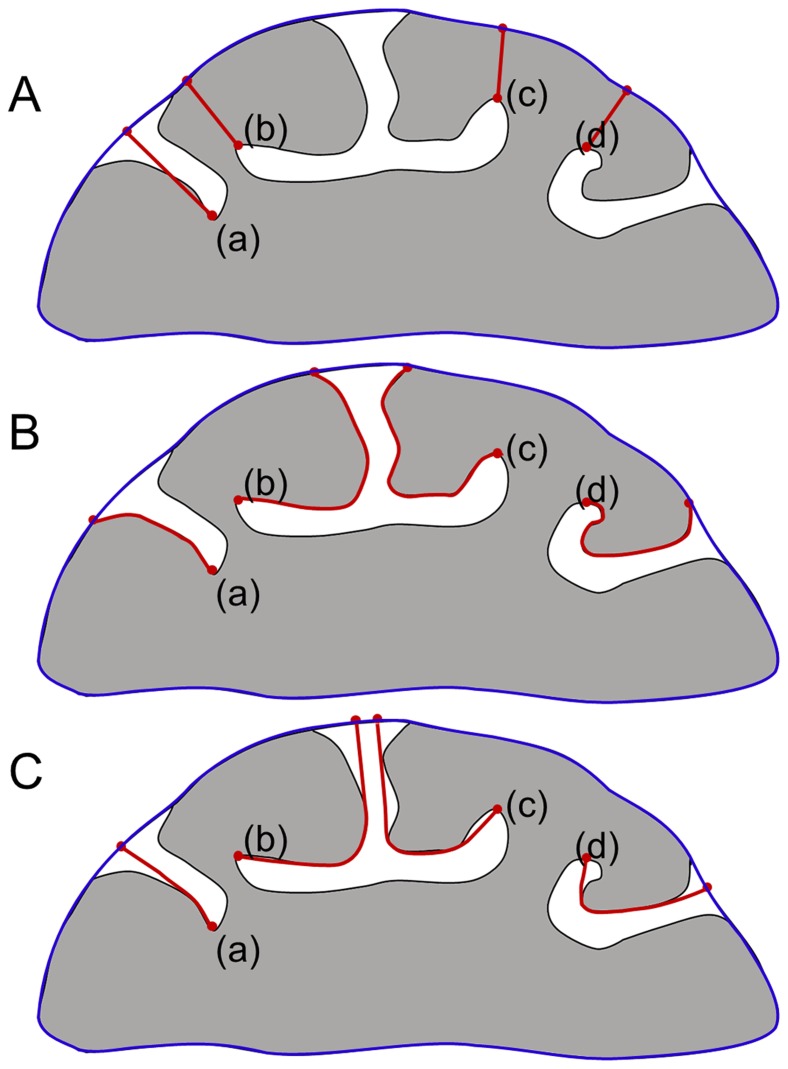
Illustration of algorithms for computing sulcal depth: (A) EUD, (B) GED, (C) ADT. The red lines illustrate the distance paths of each approach and the blue lines denote the convex hull, i.e., the seeds for depth calculations.

Various algorithms have been proposed to compute sulcal depth from 3-dimensional (3D) T1-weighted magnetic resonance images or cortical surface models [1], [4], [22]–[25]. These conventional algorithms are generally classified into two major approaches according to their definition of distance: Euclidean depth (EUD) or geodesic depth (GED), described in Fig. 1. EUD is defined as the straight line distance between a convex hull and each vertex, and GED on a mesh represents the shortest paths from a gyral region to the vertices [13], [27].

Although both EUD and GED have been used in previous neuroimaging studies and have produced clinically or methodologically significant results in brain morphology, they have some limitations reflecting sulcal geometry. Because it does not consider the degree of sulcal folding, the shortest path of EUD (Fig. 1 (a)) could result in underestimates of depth and erroneous specification of deep sulcal regions for convoluted sulci. Although GED can fully reflect the degree of sulcal folding (Fig. 1 (b)), the approach has other limitations caused by the positions of seed points and detour characteristics. In particular, it is very difficult to extract gyral regions automatically, even though they are generally defined as seed points of GED. Moreover, the results of GED vary depending on the positions of seed points. The characteristics of GED mean that, at the path around point D in Fig. 1 (b), the algorithm must make a detour along the surface instead of taking the shortest path; GED may therefore overestimate results and detect the deepest points inaccurately. Points B, C and D in Fig. 1 demonstrate extreme cases of sulci to illustrate the limitations of the two major approaches; however, these anatomical cases can be shown in human brain structures such as the Sylvian fissure. Use of these two algorithms could result in under- or overestimations of sulcal depth in aspects of methodological properties or biological meaning. To overcome these limitations and to benefit studies of brain morphology, an algorithm that reflects the geometric properties of the highly convoluted and folded structure of sulci is needed.

In this paper, we proposed a novel algorithm that is sensitive and robust, defined as the shortest path from an outer convex hull to vertices in the cortical surface following sulcal geometry (Fig. 1 (c)). This algorithm, named the adaptive distance transform (ADT), was based on Dijkstra’s algorithm [28], [29], which is a graph-searching algorithm to find the shortest path from a source. We compared the proposed method with EUD and GED in terms of its robustness and sensitivity for sulcal depth. A simulated dataset that was similar to real sulcal shapes with known deepest locations was used to analyze the robustness of the proposed method. We also investigated the difference in sensitivity between the algorithms when applied to data from patients with mild AD and control subjects.

## Materials and Methods

### Ethics Statement

Data used in the preparation of this article were obtained from the OASIS database. Written informed consent was obtained from all subjects and all studies were approved by the University’s Institutional Review Board.

### Subjects

Fifty right-handed subjects (aged 65–96 yr) were selected from the Open Access Series of Imaging Studies (OASIS) database (www.oasis-brains.org) [30]. The OASIS cross-sectional dataset has a collection of 416 subjects aged from 18 to 96, including older adults with dementia. T1-weighted magnetization-prepared rapid gradient echo images were acquired. The scans were acquired using a 1.5T Vision scanner (Siemens, Erlangen, Germany). We excluded 181 subjects for whom no clinical dementia rating (CDR) was available. The remaining subjects were divided into four groups according to CDR: very mild AD patients (CDR 0.5; *n* = 70), mild AD patients (CDR 1; *n* = 28), moderate AD patients (CDR 2; *n* = 2) and normal controls (CDR 0; *n* = 135). For this study, we selected data from all 28 mild AD patients but three of them were excluded because they failed in brain extraction (see section “Image processing”). We also selected 25 normal controls that were age- and sex-matched with the mild AD patients. The subject demographics are summarized in Table 1.

**Table 1:** Demographic characteristics of normal controls and subjects with Alzheimer’s disease.

	NC (*n* = 25)	mild AD (*n* = 25)
**Age**	74.88±7.6 (86–66)	77.76±7.1 (96–65)
**Gender**	8 males, 17 females	8 males, 17 females
**CDR**	0	1
**MMSE score**	28.56±1.6 (25–30)	21.52±3.7 (15–29)
**Years of education**	2.84±1.3 (1–5)	2.6±1.4 (1–5)

Data for age, mini–mental state examination (MMSE) score and years of education: mean ± SD (range).

### Image Acquisition

For each subject, three to four individual T1-weighted magnetization-prepared rapid gradient echo (MP-RAGE) scans were obtained on a 1.5T Vision system (Siemens, Erlangen, Germany) with the following protocol: in-plane resolution = 256 ×256 (1 mm × 1 mm), slice thickness = 1.25 mm, TR = 9.7 ms, TE = 4 ms, flip angle = 10°, TI = 20 ms, TD = 200 ms. Images were motion corrected and averaged to create a single image with a high contrast-to-noise ratio [30].

### Image Processing

T1-weighted images were registered in the ICBM 152 average template using an affine transformation and corrected for intensity nonuniformity artifacts [31], [32]. Following nonuniformity correction, brain mask was segmented using BET (brain extraction tool) [33]. The images after skull removal were then classified into white matter (WM), gray matter (GM), cerebrospinal fluid (CSF) and background using an advanced neural net classifier [34]. Hemispheric cortical surfaces were automatically extracted from each T1-weighted image using the Constrained Laplacian-based Automated Segmentation with Proximities (CLASP) algorithm, which reconstructed the inner cortical surface by deforming a spherical mesh onto the WM/GM boundary and then expanding the deformable model to the GM/CSF boundary [35], [36]. The reconstructed hemispheric cortical surfaces consisted of 40,962 vertices, each forming high-resolution meshes. The cortical surfaces were inversely transformed from the ICBM 152 template space to native space to calculate sulcal depth in native space.

### Computing the Adaptive Distance Transform

#### Local coordinates

**Figure 2:**
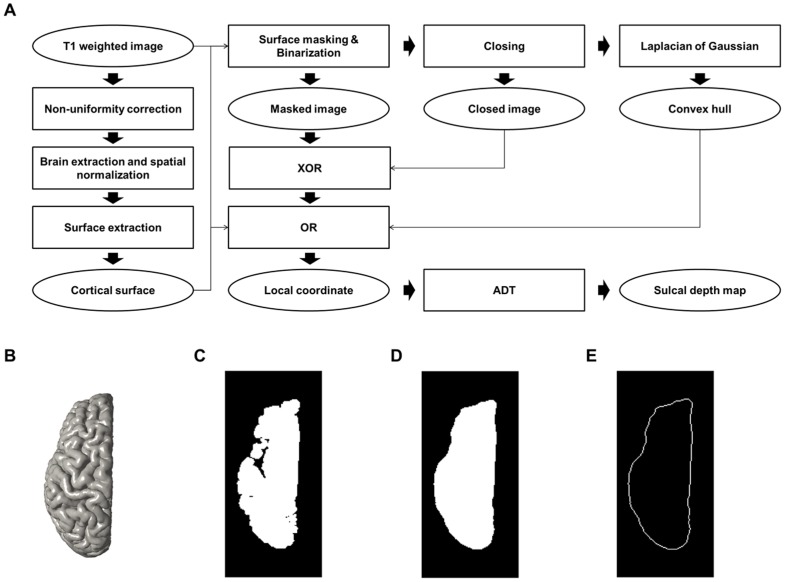
Procedures and intermediate results of image processing. Block diagram of image processing and local coordinate construction steps (A) and intermediate results: (B) cortical surface, (C) masked image, (D) closed image, (E) convex hull.

The implementation of ADT on the cortical surface requires a convex hull and local coordinates. Local coordinates are 3D Cartesian coordinates between the convex hull and the cortical surface model [22]. We converted resolution of T1 weighted images to 1 mm isotropic voxel size because anisotropic voxel resolution could result biased sulcal depth. Searching and calculating the distance using ADT to the neighbor nodes in anisotropic local coordinates were lopsided by directionally dependent distance between nodes. Since it would become a serious problem that spatial orientation of sulci would affect measuring sulcal depth, we converted local coordinates to isotropic level. Converted volume images were then constructed the convex hull on volume images that had been used in previous studies [1], [13]. We made masked volume images isolating the voxels inside the cortical surface. Each masked image was binarized and we performed a 3D morphological closing operation with a 10 mm spherical kernel known as large enough radius (see Supplementary Table S1) that could fill the sulci in previous studies [1], [13]. The Laplacian of a Gaussian mask was used to construct a convex hull from the closed image. On volume images, the local coordinates were represented in voxel dimensions. We used the closed volume image and cortical surface to construct the local coordinates. As with the masked image, the voxels inside the surface were eliminated from the closed image. The remaining voxels became the local coordinates, which had a 1 mm isotropic level according to our dataset. Due to short distance between vertices of cortical surface (in our data, mean distance is 0.87±0.03 mm), we divided the local coordinates to the 0.5 mm level to improve the accuracy of ADT. Lower level of local coordinates seemed to generate precise paths and depth value but it was practically inefficient because of efficiency-accuracy trade-off. The lower level of coordinates we sampled, the higher accuracy we could obtain. The improvement of accuracy, however, was inefficient, and was at the cost of much longer computation time (see Supplementary Figure S1 and S2). Hence, we concluded that the increase in computation time through the low level of local coordinates was ineffective and finally we chose 0.5 mm level. All the procedures are described in Fig. 2.

**Figure 3:**
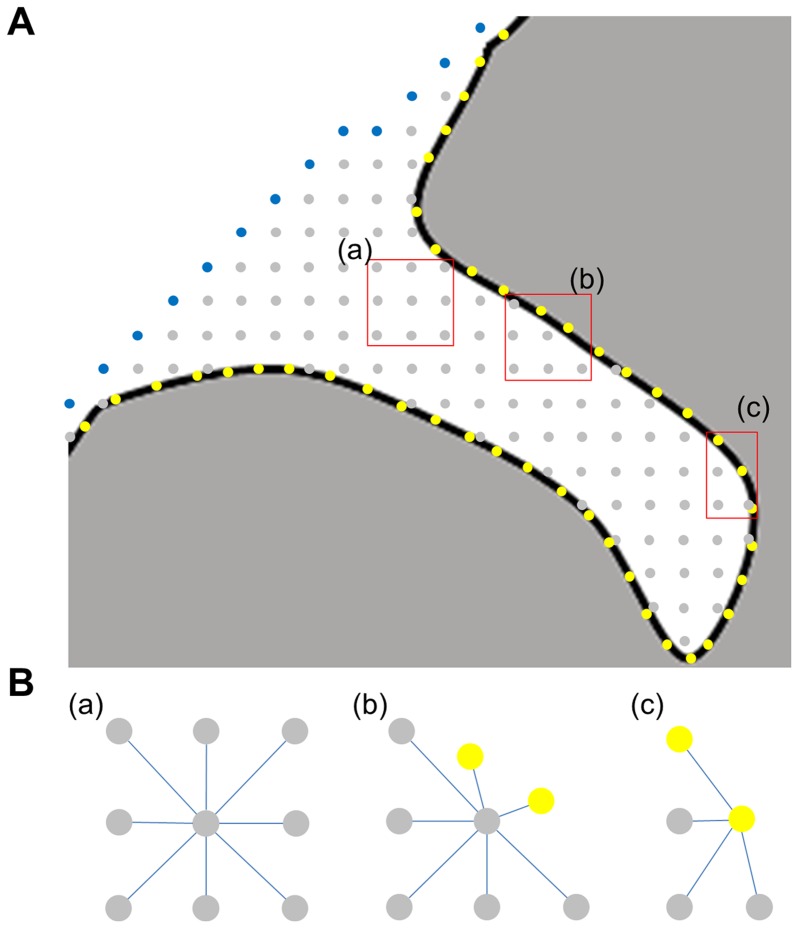
An illustrative figure to show the local coordinates. (A) Local coordinates, (B) “Accepted” nodes and their “Considered” nodes with temporary edges. Gray nodes are in the voxel space between the convex hull (blue nodes) and the cortical surface (yellow nodes). The edge cost 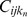 is indicated by the length of the blue line.

The constructed local coordinates, which are the same as those in Kao et al. [22], do not include the convex hull and the cortical surface. However, our purpose in implementing the ADT algorithm is to map the sulcal depth on the cerebral sulci and the path of ADT starts from the convex hull. We modified the local coordinates used in Kao’s method, combining them with the convex hull and the vertices of the cortical surface. These reconstructed local coordinates are used as the input to ADT (Fig. 3 (a)).

#### Adaptive distance transform

The basic idea of ADT is to apply Dijkstra’s algorithm to local coordinates [28]. Dijkstra’s algorithm computes the minimum cost of reaching any node on a network, producing the shortest path. For a rectangular network, the minimum total cost 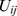 of reaching the node 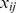 is the sum of the edge path cost 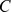 and the minimum 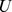 of neighbors [29]:
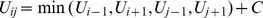
(1)


To find the minimum total cost, the algorithm separates nodes into three classes: “Far” (no information about 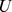), “Accepted” (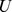 has been computed) and “Considered” (the neighborhood of “Accepted”). The algorithm changes “Considered” 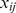 into the “Accepted” set and its “Far” neighbors into the “Considered” set. All nodes on the network were changed to the “Accepted” set according to Eq. [1].

Our approach is different from the conventional Dijkstra’s algorithm, which has prior information about edges and costs. Because the points of local coordinates do not have any information about “Considered” nodes or costs around “Accepted” nodes, we searched temporary “Considered” nodes and calculated a temporary cost around “Accepted” nodes. Each node located inside the 26-neighborhood of an “Accepted” node is classified as a “Considered” node and the Euclidean distance between the “Accepted” node and each “Considered” node becomes a cost (Fig. 3 (b)). For ADT, the modified Eq. [1] is
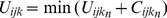
(2)


The 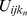 denotes the minimum total cost of the *n*th neighborhood of 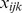 from the convex hull (starting nodes) and 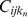 denotes the cost between 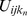 and 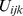. The detailed procedure of the ADT algorithm is as follows.

1. Set the 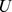 of local coordinates to infinity and the convex hull (“Accepted”) to 0.
2. Find an “Accepted” node 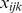.
3. Search the temporary “Considered” nodes around the “Accepted” node selected via (2) and calculate 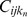.
4. Apply Eq. [2] to the “Accepted” node.
5. Repeat (2) through (4) for all points in the local coordinates until no 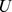 changes any further.

### Computing EUD and GED

We implemented and applied EUD and GED to our dataset to compare their performance with that of our algorithm [13], [27]. In simple terms, the EUD was calculated as the shortest distance between the convex hull and the vertices of the cortical surface. All vertices of gyral regions were set to zero and the Eikonal equation was solved to measure GED in vertices of sulcal regions from gyral regions.

### Simulation Data

**Figure 4:**
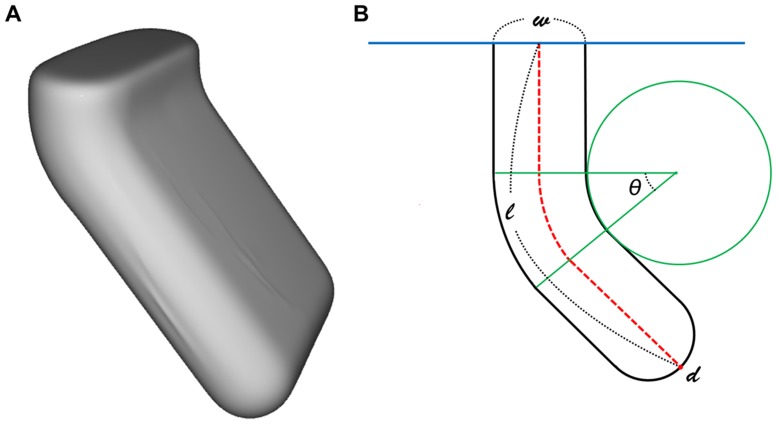
Extracted simulation data using the modified CLASP algorithm (A), and its diagram (B). The components managing the morphometry of the simulation data are shown in (B). 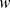 is sulcal width; 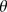 is the folding degree from an outside point located on a line parallel to the seed of distance (blue line); and 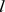 is the length of the medial line (red dotted line) with its end point defined as the deepest point (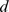).

The locations of the deepest points of sulci can be defined using a sulcal depth map, but they have spatial variability according to the measurement algorithm used. For complicated sulci, conventional algorithms for sulcal depth might detect erroneous locations because of their methodological properties, and thus could produce under- or overestimated results. To analyze the robustness, we therefore created a simulation dataset that is similar to real sulcal shapes; in the simulations, the location of the deepest point is easily defined (Fig. 4 (a)). We assumed that three components mainly contribute to sulcal morphology: sulcal width (*w*), folding degree (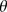) and length (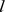) of the medial line of the sulcus. Fifty-six simulated sulci were designed by changing these components from 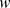 = 10,…,50, 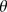 = 0,…,90 and 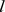 = 120,…150 (Fig. 4 (b)).

First, the simulation dataset was constructed as a volume image, and then the modified CLASP algorithm [35], [36] was applied to extract surface models of the simulation dataset consisting of 20,480 discrete triangular elements (10,242 vertices). Finally, we defined the deepest point (*d*) as an end point of a medial line and the measured length of the medial line as the depth of the simulation dataset (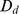).

### Data Analysis

We analyzed group differences of whole-brain mean sulcal depth using an analysis of covariance (ANCOVA) with intracranial volume, age and sex as covariates to compare the algorithms in terms of sensitivity. The area under receiver operating characteristic (ROC) curve (AUC) for normal control and mild AD groups were calculated as a measure of performance for classifying subjects as control or mild AD. These statistical measures to capture the change in sulcal depth were used as the level of sensitivity.

Even in the same sulcus, deep sulcal regions have different locations defending on algorithms. For instance, point D in Fig. 1 (c) indicated the deepest point measured by ADT but the location of the deepest point could be different by properties of algorithms. For this reason, we used the simulation dataset to compare the algorithms’ robustness and ability to cope with intersubject variability in deep sulcal regions. To evaluate the robustness of each algorithm, we compared the normalized differences between 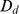 and the maximum depth value (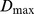) estimated by each algorithm in the simulation dataset. The differences in simulation data were categorized and averaged by components.

## Results

### Clinical Data

**Figure 5:**
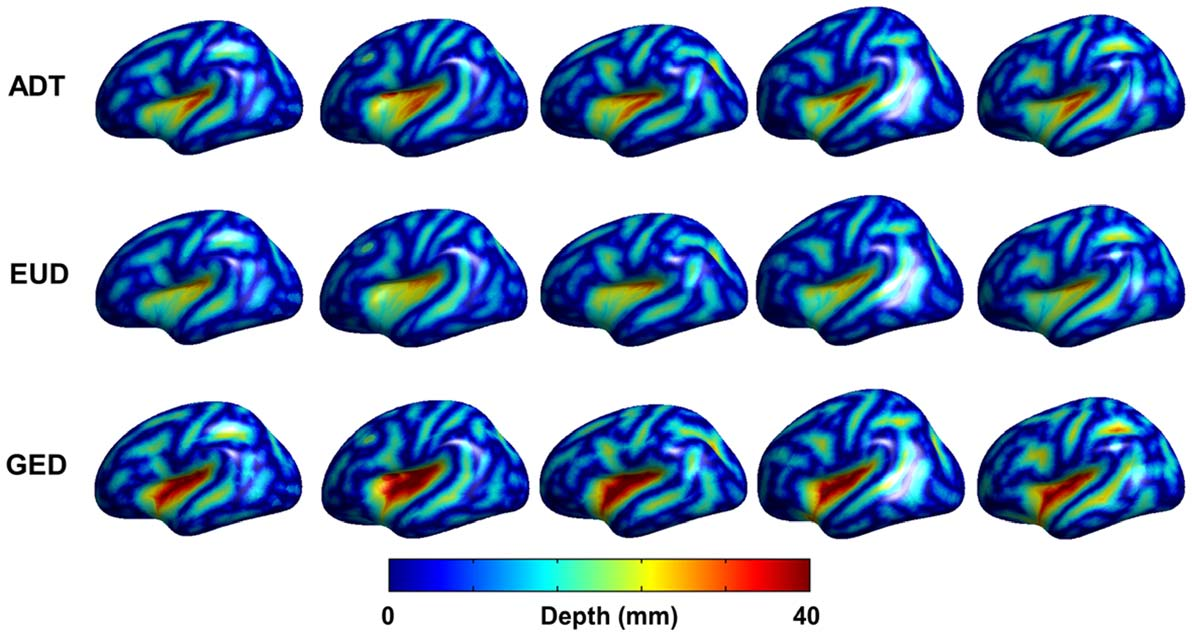
Sulcal depth maps on cortical surface of 5 randomly selected clinical dataset are inflated to display deep sulcal region.

**Figure 6:**
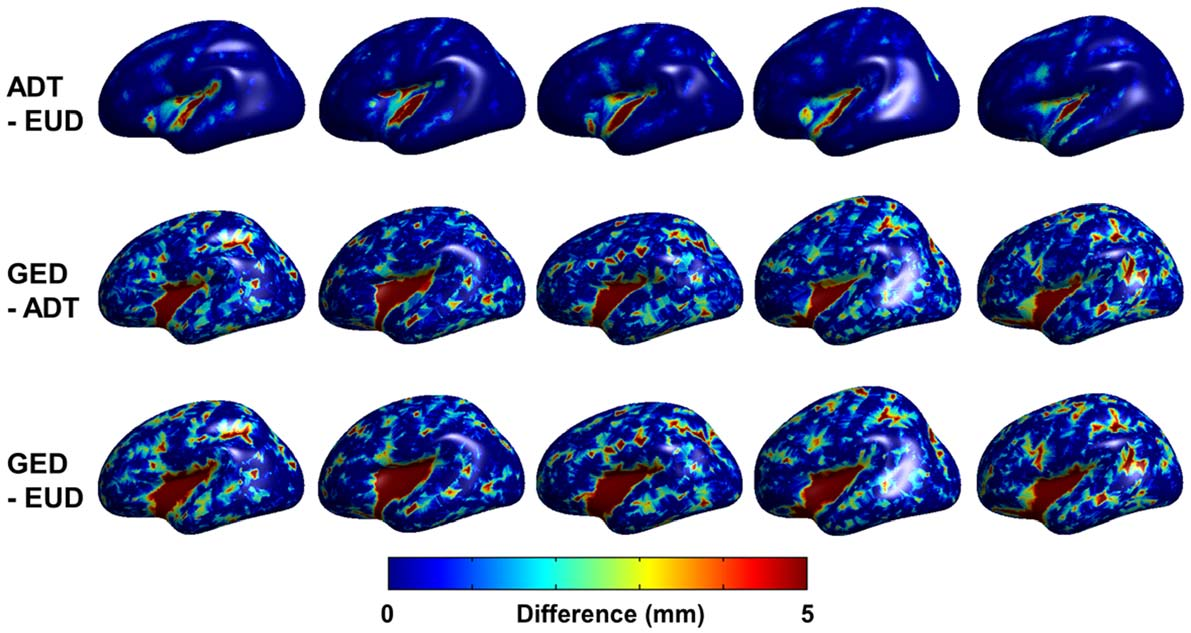
An example of the differences among the algorithms in the 5 subjects.

**Figure 7:**
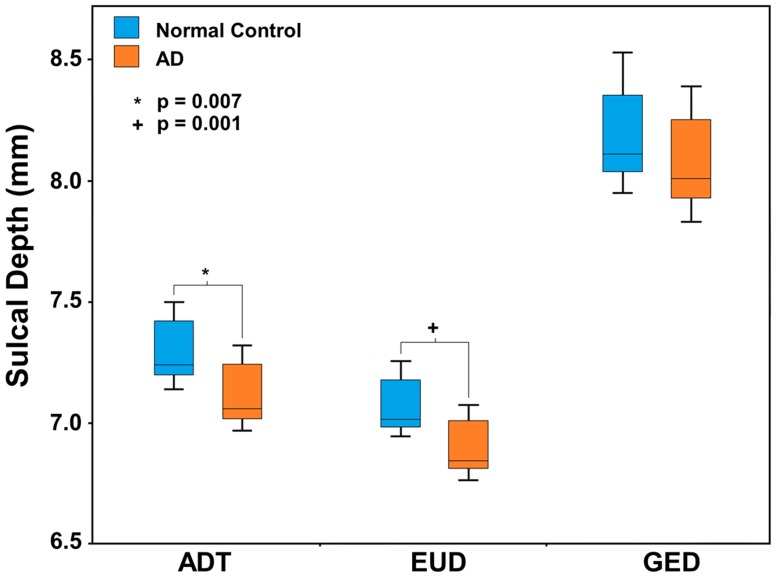
The group difference of mean sulcal depth. Box plot displaying the data distributions and differences in average mean sulcal depth between groups of normal controls and patients with mild AD measured using ADT, EUD and GED.

**Figure 8:**
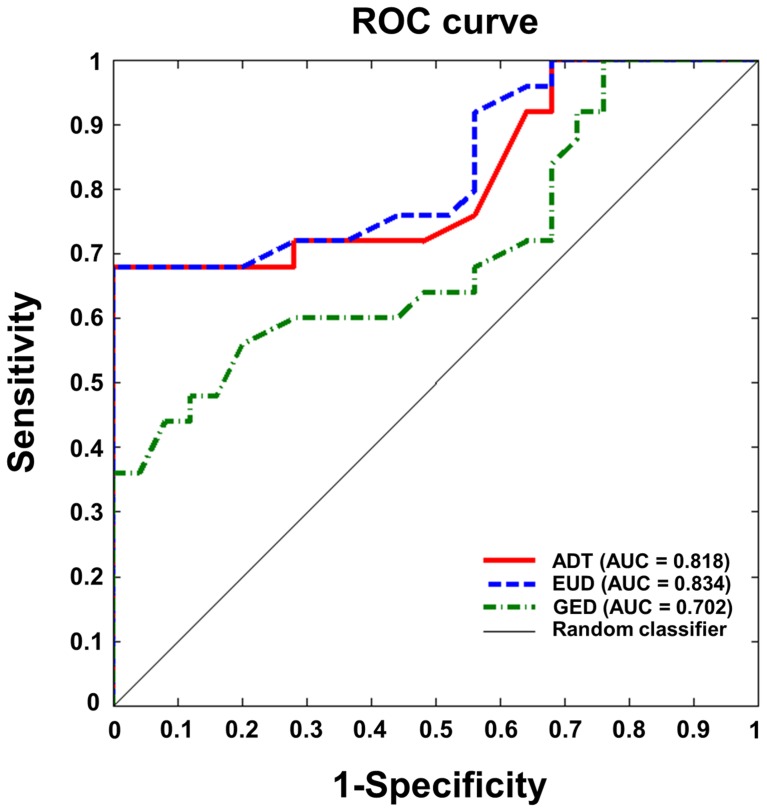
ROC curve results. ROC curves are plots of sensitivity and specificity of algorithms for distinguishing normal controls from patients with mild AD. AUCs are added in the figure.

Individual sulcal depth map of all three algorithms are shown in Fig. 5 and the difference among the algorithms are shown in Fig. 6. The sensitivity of ADT was compared with that of EUD and GED using the clinical dataset. Comparisons of whole-brain mean sulcal depth for normal control subjects and mild AD patients using the three algorithms are shown in Fig. 7. ADT showed significant differences in mean depth between control subjects and patients with mild AD (*p* = 0.007). Sulcal depth measured using EUD was significantly shallower for patients with mild AD than for controls (*p* = 0.001). Although GED for controls trended slightly higher than for mild AD subjects, the effective size of the group difference did not achieve statistical significance (*p* = 0.066). The ROC curves for all three algorithms are shown in Fig. 8. The AUC values were as follows: ADT 0.818, EUD 0.834 and GED 0.702. A higher AUC value indicates that the algorithm more clearly distinguishes patients from normal controls.

### Simulation Data

**Figure 9:**
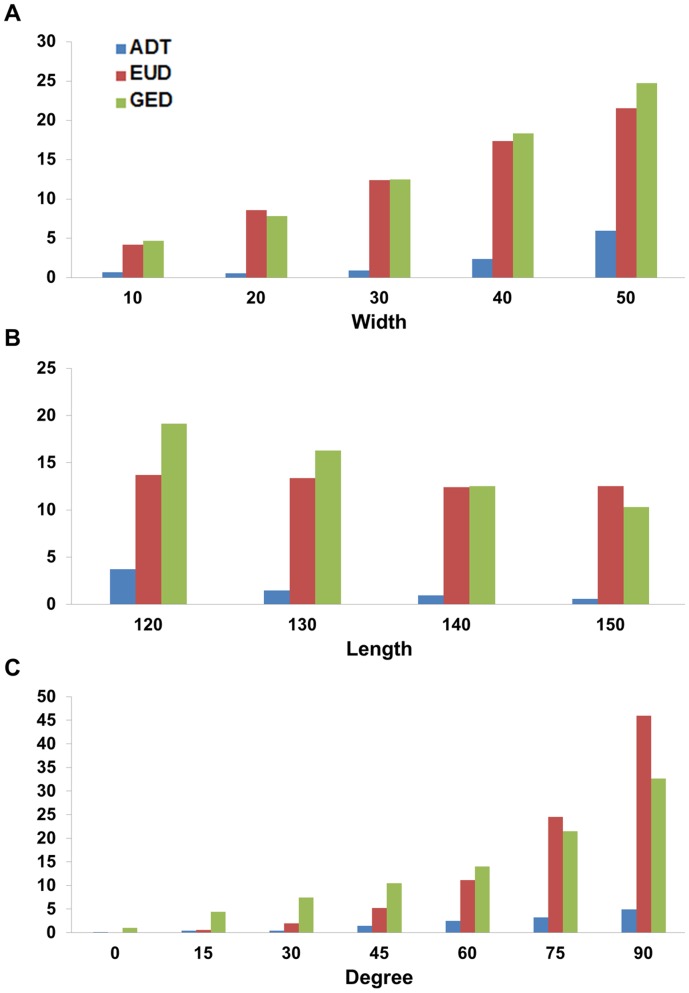
Average normalized difference of each algorithm in the simulation dataset. The normalized difference is measured using the following difference rate equation: 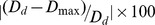.

The performance-normalized differences for the 56 simulated sulci and a range of each component are plotted in Fig. 9. Within all of the ranges of components shown, the ADT algorithm exhibited lower difference rates (0.11–5.98%) than EUD (0.01–34.98%) and GED (1.12–28.59%). In general, the difference consistently increased in all three algorithms as the values of components increased. However, the difference rate for ADT increased only slightly whereas EUD and GED produced dramatically higher difference rates.

## Discussion

We have proposed a novel algorithm for measuring sulcal depth, named ADT, to overcome the limitations of the EUD and GED. The basic idea of ADT is to apply Dijkstra’s algorithm to local coordinates. ADT produced low and constant normalized differences in the simulation dataset for all three components associated with sulcal complexity. Its results for sensitivity were similar to those from EUD but better compared with GED. Consequently, ADT was demonstrated to be a robust and sensitive measure for the sulcal depth compared with existing approaches.

### Robustness against Variable Sulcal Geometry

In previous studies, deep sulcal regions were used as sulcal landmarks because of their robustness against sulcal geometry [10], [11], [14], [15], [22], [23], [37]. Although this robustness could be compared according to each algorithm in terms of the location of the deepest points in the sulci, there is no gold standard for deepest points. Therefore, the simulation dataset was designed to analyze this robustness. The normalized difference of ADT was lower and more consistent than that of the other algorithms. In particular, the ADT algorithm had a low effect and constant normalized difference rate on the component “degree” whereas EUD and GED produced extremely large differences. The component “degree” reflects how complicated and convoluted a sulcus is. In other words, ADT appears to be independent of the sulcal complexity and has superior robustness to other algorithms. This result can be explained by ADT’s property of reflecting sulcal geometry; that is, it can define deep sulcal regions even though sulcal shape is arbitrary. The robustness of sulcal depth against sulcal variability could influence the extraction of deep sulcal landmarks such as sulcal pits, fundi and lines in highly convoluted and folded sulcal structures. We therefore conclude that ADT is a more powerful method for brain morphological studies than EUD or GED in terms of robustness.

### Sensitivity to Morphological Changes in the Brain

Cortical atrophy on the pial surface decreases the sulcal depth. Many previous studies used EUD or GED algorithms to investigate age- or disease-related changes in sulcal depth as sensitive features of cortical atrophy [1], [3], [8], [9], [24], [25], and reported significant differences between control subjects and patients with AD [1]. Shallower depth patterns have been found in AD patients than in control subjects. Our results corroborate the findings of significant differences in sulcal depth between control subjects and patients with mild AD. In terms of distinctions between controls and mild AD patients, the AUC analysis suggests that ADT and EUD algorithms are “good” tests but GED is only “fair”. Our results show that ADT offers a sensitive measure for computing sulcal depth. The ADT and EUD algorithms were similar in terms of sensitivity, with evidently similar results between the two algorithms. There are two reasons for this. First, in pial surfaces, because the sulcus is a very narrow structure and most sulci have simple shapes, the paths of each algorithm showed small differences, thus little affecting the algorithms’ sensitivities. Second, the mean sulcal depth of all vertices can distort the effect of different paths. Gyral regions and sulcal walls were found to give no information for calculating the mean sulcal depth. Although recent researches in brain morphology have used vertex-wise or lobar−/regional-level comparison utilizing sphere-to-sphere warping surface registration [38], [39], this surface registration used depth potential map [40] computed by solving a time independent Poisson equation as feature field to match sphere to sphere. It could be biased in this study, comparisons of 3 algorithms measuring sulcal depth, because the pattern of depth potential map was different to all 3 algorithms. Eventually, it was not fair comparison involved surface registration based on depth potential to lobal−/regional- analysis of sulcal depth. It was also not clarified if we analyzed sulcal depth using vertex-wise comparison, the statistically significant vertices were really significant sulcal region. Because gyral region, sulcal wall and deep sulcal region were not defined even in surface template, it could confuse us to interpret results. Furthermore, some previous studies [1], [3] defined sulcal regions setting the threshold of sulcal depth value by eliminating the effects of regions with no information. However, in this study, differences in depth values calculated by the three algorithms made it difficult to set sensible threshold values, as in previous studies. To compensate the limited results, we showed the individual sulcal depth maps of the 3 measurements in Fig. 5. We also showed an example of the differences among the algorithms in the 5 subjects in Fig. 6. In future work, the sensitivity to sulcal depth may be needed for more exact distinctions between groups using parcellation of deep sulcal region and unbiased registration methods.

### Adaptive Distance Transform Using Modified Local Coordinates

The local coordinates between the convex hull and the cortical surface used in this study were constructed based on Kao’s method. Indeed, ADT is similar to the algorithm proposed in Kao et al. [22]. However, we computed sulcal depth using Dijkstra’s algorithm with reconstructed local coordinates that combined the vertices of the cortical surface model whereas Kao solved the Eikonal equation on local coordinates that did not include vertices. The difference of local coordinate could produce different results because ADT computes sulcal depth on vertices directly while Kao performs linear interpolation to vertices. Besides, it should be noted that difference between Dijkstra’s algorithm and Eikonal equation could affect paths of sulcal depth. The basic concept of these two algorithms is same for computing shortest paths on a network. Dijkstra’s algorithm computes paths along the discrete network edges but Eikonal equation approximates paths underlying partial differential equation. This difference between Dijkstra’s algorithm and Eikonal equation could result in similar or little bit different paths. Although Eikonal equation makes more fluid path than that of Dijkstra’s algorithm, it is more intuitive that node to node path generated by Dijkstra’s algorithm in that Dijkstra’s algorithm could calculate real depth on local coordinates instead of approximated depth.

### Conclusions

This study demonstrates the potential of ADT as an alternative method for measuring sulcal depth, in terms of improved robustness and sensitivity. We have shown that ADT is more robust than the conventional approaches and has enough sensitivity to identify cortical atrophy and diagnose AD. The ADT algorithm should be used for morphometric and clinical analysis to generate better results.

## Supporting Information

Figure S1 **Computation time with division level in five individual subjects.** X-axis indicates division level and y-axis means computation time for ADT.
(TIF)

Figure S2 **Mean sulcal depth changed by division level in five individual subjects.**
(TIF)

Table S1Closed volume(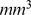) changed by kernel size in three AD subjects.(DOCX)
