# Supplementary figures and images for: Automated Sulcal Depth Measurement on Cortical Surface Reflecting Geometrical Properties of Sulci
Source: PLoS One. 2013 Feb 13;8(2):e55977. doi: 10.1371/journal.pone.0055977 (PMC3572156; doi:10.1371/journal.pone.0055977)

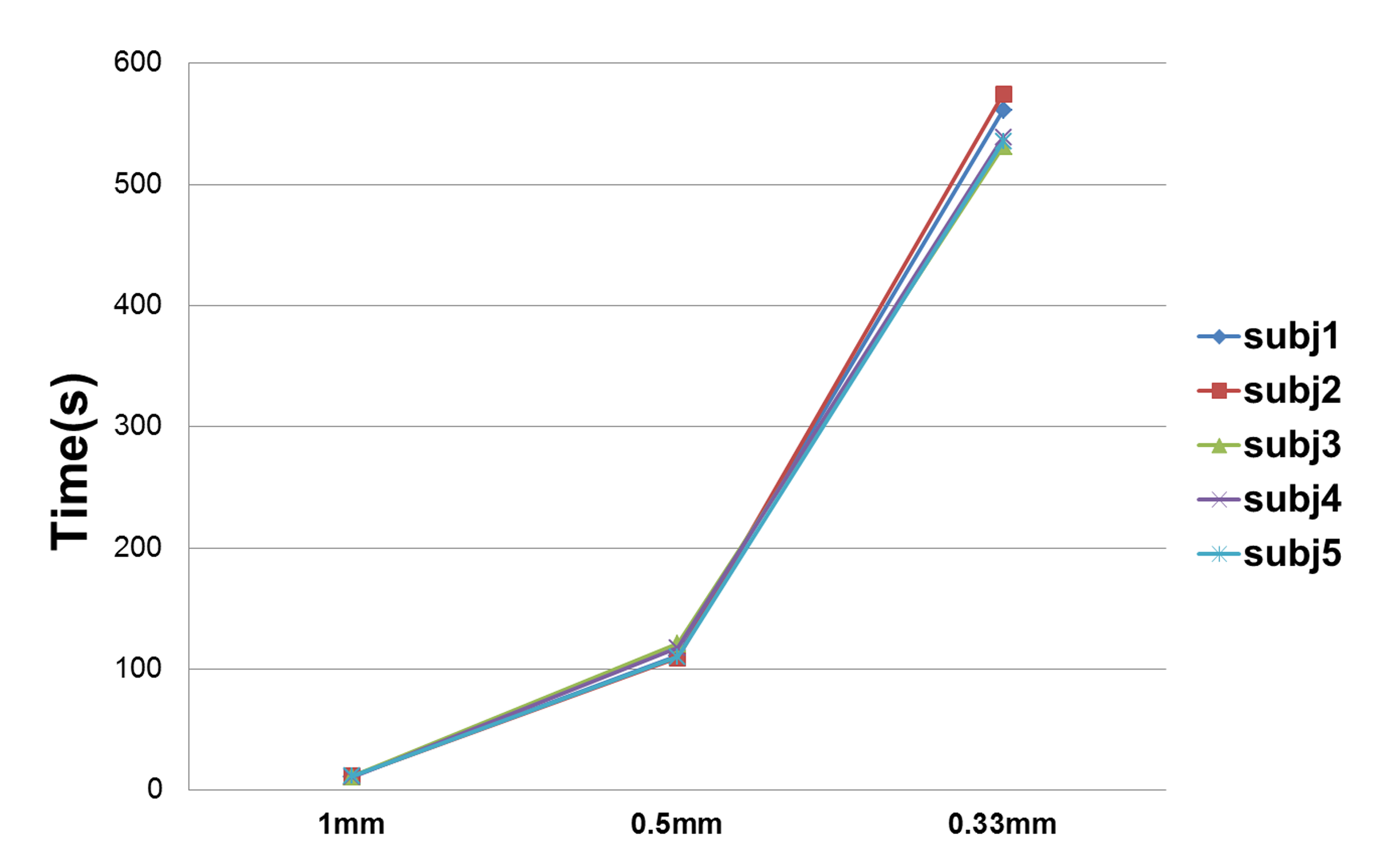

Supplement: Figure S1 — Computation time with division level in five individual subjects. X-axis indicates division level and y-axis means computation time for ADT. (TIF) [file pone.0055977.s001.tif]

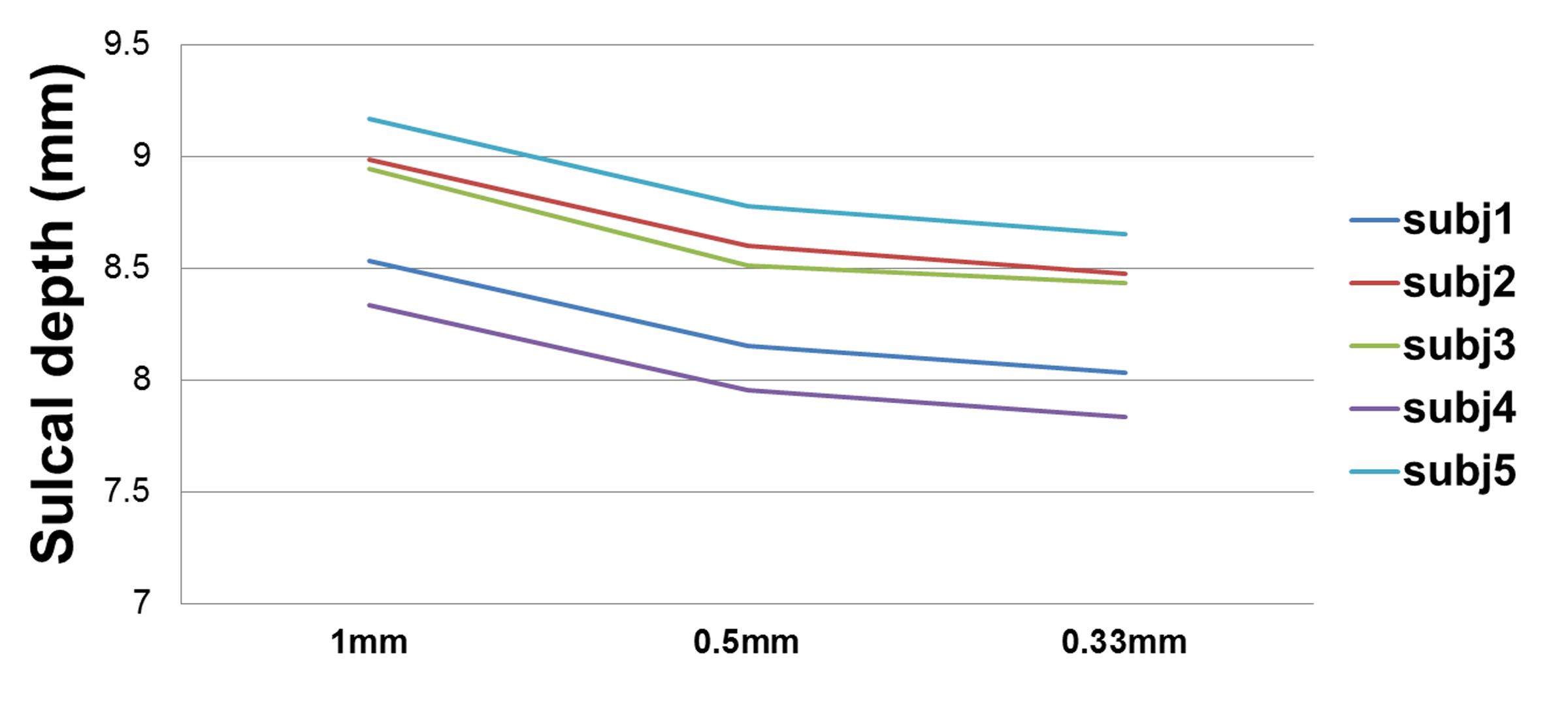

Supplement: Figure S2 — Mean sulcal depth changed by division level in five individual subjects. (TIF) [file pone.0055977.s002.tif]
