# Supplementary material for: Automated Sulcal Depth Measurement on Cortical Surface Reflecting Geometrical Properties of Sulci
Source: PLoS One. 2013 Feb 13;8(2):e55977. doi: 10.1371/journal.pone.0055977 (PMC3572156; doi:10.1371/journal.pone.0055977)
Supplement: Table S1 — Closed volume() changed by kernel size in three AD subjects. (DOCX) [file pone.0055977.s003.docx]

|  | Kernel size | | | |
| --- | --- | --- | --- | --- |
|  | **10mm** | **15mm** | **20mm** | **25mm** |
| AD subject #1 | 531046 | 538238 | 544532 | 549116 |
| AD subject #2 | 533741 | 540280 | 545436 | 548975 |
| AD subject #3 | 556121 | 563108 | 567790 | 571297 |
